# *BDNF* Val66Met Genotype, DNA Methylation, mRNA, and Protein Levels as Potential Blood-Based Biomarkers for Dementia and Cognitive Decline

**DOI:** 10.3390/ijms26188987

**Published:** 2025-09-15

**Authors:** Lucija Tudor, Alja Videtic Paska, Marcela Konjevod, Nikola Balic, Matea Nikolac Perkovic, Suzana Uzun, Barbara Vuic, Tina Milos, Gordana Nedic Erjavec, Ninoslav Mimica, Katarina Kouter, Nela Pivac, Dubravka Svob Strac

**Affiliations:** 1 Laboratory for Molecular Neuropsychiatry, Division of Molecular Medicine, Ruder Boskovic Institute, 10000 Zagreb, Croatia; marcela.konjevod@irb.hr (M.K.); nikola.balic@irb.hr (N.B.); matea.nikolac.perkovic@irb.hr (M.N.P.); barbara.vuic@irb.hr (B.V.); tina.milos@irb.hr (T.M.); gordana.nedic.erjavec@irb.hr (G.N.E.); 2 Centre for Functional Genomics and Biochips, Institute of Biochemistry and Molecular Genetics, Faculty of Medicine, University of Ljubljana, 1000 Ljubljana, Slovenia; alja.videtic@mf.uni-lj.si; 3 Department for Biological Psychiatry and Psychogeriatry, University Psychiatric Hospital Vrapce, 10000 Zagreb, Croatia; suzana.uzun@gmail.com (S.U.); ninoslav.mimica@bolnica-vrapce.hr (N.M.); 4 School of Medicine, University of Zagreb, 10000 Zagreb, Croatia; 5 Institute of Microbiology and Immunology, Faculty of Medicine, University of Ljubljana, 1000 Ljubljana, Slovenia; katarina.kouter@mf.uni-lj.si; 6 University of Applied Sciences Hrvatsko Zagorje Krapina, 49000 Krapina, Croatia; npivac@irb.hr

**Keywords:** BDNF, Val66Met polymorphism, DNA methylation, gene and protein expression, dementia, MCI

## Abstract

Brain-derived neurotrophic factor (BDNF) plays a crucial role in cognitive functions and dementia. In individuals with mild cognitive impairment (MCI) and dementia, we have investigated *BDNF* Val66Met genotype distribution, peripheral *BDNF* DNA methylation, mRNA and protein levels, and cognitive performance using the Mini-Mental State Examination (MMSE) and Clock Drawing Test (CDT). Lower BDNF_IV1 methylation had predictive value for dementia. Patients with mild-to-moderate dementia had lower levels of BDNF_IV2 methylation, whereas patients with severe dementia had higher levels than the MCI group, while BDNF_IV2 methylation positively correlated with CDT scores. An insignificant decline in BDNF mRNA levels in dementia patients positively correlated with significantly lower BDNF plasma levels, especially pronounced in severe dementia patients. BDNF mRNA and protein levels were positively correlated with CDT and MMSE scores, respectively. BDNF Val66Met polymorphism was associated with methylation of the BDNF_IX amplicon, but not with methylation in BDNF promoters I and IV, peripheral BDNF gene and protein expression, MMSE and CDT scores, or dementia. Methylation at the BDNF Val66Met site was positively correlated with overall BDNF_IX methylation and methylation at 5 BDNF_IX CpG loci but negatively correlated with methylation of BDNF_IV1, BDNF_IV3, and BDNF_I1 amplicons. Further studies should evaluate the translational potential of these peripheral BDNF-based biomarkers for dementia.

## 1. Introduction

Dementia is characterized by a progressive decline in cognitive functions, particularly memory and thinking, which impairs the individual’s ability to perform daily activities and is often accompanied by alternations in behavior and personality [1]. Approximately 57 million individuals worldwide are currently diagnosed with dementia, with nearly 10 million new diagnoses arising annually, making it one of the leading global causes of mortality [2]. Most common dementia causes include Alzheimer’s disease (AD), vascular dementia, frontotemporal dementia, and Lewy body dementia [3]. Individuals with mild cognitive impairment (MCI) can still independently manage their daily activities; however, they have a higher risk of developing dementia [4].

Compelling evidence indicates a role of brain-derived neurotrophic factor (BDNF) in cognitive functions and dementia [5,6]. This neurotrophin impacts neuronal survival, dendritic and axonal growth, synaptogenesis, and the mechanisms of long-term potentiation, which are the basis of learning and memory [7,8]. In accordance with its pro-synaptic functions, *BDNF* mRNA and BDNF protein levels are significantly reduced in the hippocampus and associative cortex of AD patients, corresponding to synapse loss and cognitive decline [6,9]. Reduced BDNF levels have already been observed in the MCI stage and have been found to correlate with cognitive function [10]. The transcription of the *BDNF* gene, located on chromosome 11, is regulated by various activity-responsive promoters, making it susceptible to epigenetic modulation. Hypermethylation in promoters I and IV has consistently been observed in the brain tissue of post-mortem AD patients and in peripheral blood, where it is associated with diminished cognitive performance [11,12]. Although peripheral *BDNF* promoter methylation has been proposed as a minimally invasive prodromal biomarker, studies report significant heterogeneity among different cohorts and investigated CpG sites [13]. Genetic variation also modulates BDNF levels. The most investigated *BDNF* functional single-nucleotide polymorphism is Val66Met (rs6265), which substitutes valine for methionine in the BDNF pro-domain, impairing activity-dependent BDNF secretion and consequently affecting hippocampal structure and episodic memory performance [14]. Emerging evidence indicates a bidirectional interaction between *BDNF* Val66Met polymorphism and DNA methylation: the Met allele may predict hypo- or hypermethylation within exon IX and at distant promoters, potentially shaping individual susceptibility to cognitive decline [15,16]. Despite these insights, comprehensive studies that concurrently investigate region-specific *BDNF* methylation, peripheral *BDNF* mRNA and protein expression, *BDNF* rs6265 genotype, and neurocognitive profiles from MCI to dementia patients remain rare. Our initial findings from the Croatian cohort suggest elevated peripheral *BDNF* expression in MCI compared to AD, but the correlations among methylation status, genotype, and cognition remain unresolved [17].

Therefore, this study investigates *BDNF* Val66Met genotypes, peripheral methylation at six *BDNF* regions, and *BDNF* mRNA and protein levels and correlates these variables with cognitive performance of individuals with MCI and dementia. By examining how *BDNF* genetic and epigenetic regulation shape BDNF expression and consequently cognitive abilities, we aim to elucidate pathogenic mechanisms underlying cognitive decline and assess the translational potential of peripheral BDNF-based biomarkers for dementia.

## 2. Results

### 2.1. Analysis of Participant’s Demographic and Clinical Data

This study included a total of 406 (177 female) participants: 251 individuals with MCI (mean age 67.34 ± 5.63, ranging from 56 to 89 years) and 155 persons with dementia (mean age 69.88 ± 6.30, ranging from 58 to 89 years). Demographic and clinical data are listed in Table 1. As expected, subjects with dementia were significantly older than subjects with MCI (*p* = 0.001) and had a higher proportion of female participants (*p* = 0.011). Moreover, dementia patients had significantly lower scores on Mini-Mental State Examination (MMSE) and Clock Drawing Test (CDT) scales. The clinical assessment indicated that most of the dementia patients were in the group with mild to moderate dementia, while 31 of them had severe dementia.

### 2.2. Methylation Analysis

**Figure 1:**
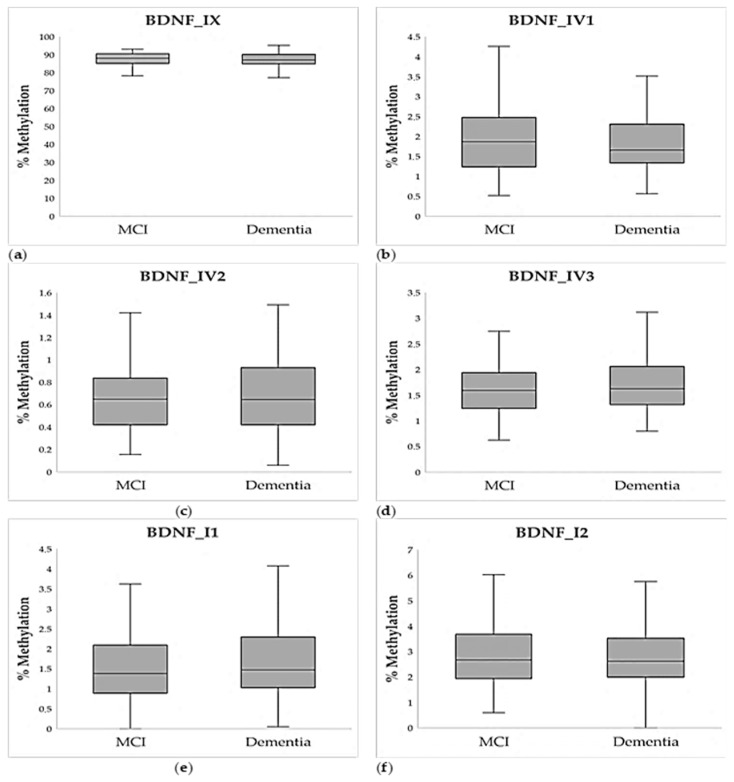
Average percent of methylation of six chosen amplicons of the *BDNF* gene in individuals with MCI (N = 86) and dementia (N = 114): (**a**) Amplicon BDNF_IX, (**b**) Amplicon BDNF_IV1, (**c**) Amplicon BDNF_IV2, (**d**) Amplicon BDNF_IV3, (**e**) Amplicon BDNF_I1, (**f**) Amplicon BDNF_I2. Data are shown as a boxplot diagram. The central box represents the interquartile range, the middle line represents the median, and the vertical line extends from Q1—1.5*IQR and Q3 + 1.5*IQR.

Methylation analysis in a subset of a total of 200 patients (114 patients with dementia, of whom 31 had severe cognitive impairment, and 86 patients with MCI) revealed that the differences in methylation of 6 chosen regions in *BDNF* promoters and exon IX were not statistically significant between individuals with dementia and MCI (Figure 1, Supplementary Table S2).

**Figure 2:**
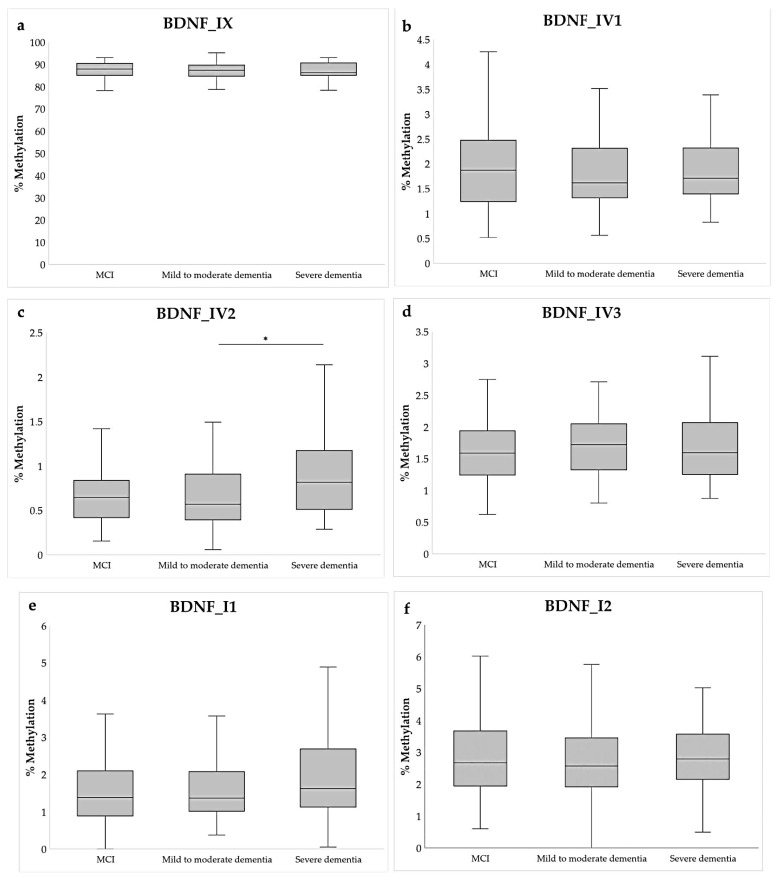
Average percent of methylation of six chosen amplicons of the *BDNF* gene in individuals with MCI (N = 86) and mild-to-moderate dementia (N = 83) and severe dementia (N = 31): (**a**) Amplicon BDNF_IX, (**b**) Amplicon BDNF_IV1, (**c**) Amplicon BDNF_IV2, (**d**) Amplicon BDNF_IV3, (**e**) Amplicon BDNF_I1, (**f**) Amplicon BDNF_I2. Data are shown as a boxplot diagram. The central box represents the interquartile range, the middle line represents the median, and the vertical line extends from Q1—1.5*IQR and Q3 + 1.5*IQR. * *p* < 0.05, post hoc Dunn test.

However, when subdividing the participants into those with MCI, mild-to-moderate dementia, and severe dementia, significant differential methylation was observed in the *BDNF_*IV2 amplicon (Kruskal–Wallis test), specifically between mild-to-moderate dementia and severe dementia (*p* = 0.013; post hoc Dunn test), and marginally between MCI and severe dementia (*p* = 0.053; post hoc Dunn test) (Figure 2, Supplementary Table S3). Although the overall methylation of this region was small (<1%), patients with mild-to-moderate dementia had lower *BDNF_*IV2 DNA methylation than MCI, but patients with severe dementia had higher levels of DNA methylation compared to both groups.

Hierarchical logistic regression was used to test the effect of age and sex as predictors of dementia, in addition to 6 tested *BDNF* amplicons. Two models have been constructed: a base model with age and sex as only predictors and the test model with added average percent of methylation of all 6 amplicons (Table 2). Collinearity between amplicons has been tested and resulted in acceptable VIF values between 1.1 and 2.1. As expected, logistic regression showed that female sex and older age are significant predictors of dementia, but there was no significant correlation between methylation in tested amplicons and age (data available on request). Moreover, using this model, lower methylation in BDNF_IV1 also had significant predictive value for dementia (*p* = 0.009; Table 2), although lower BDNF_IV1 methylation in dementia patients only showed a trend when participants were compared directly (Figure 1, Supplementary Table S2).

Individual CpGs in tested *BDNF* amplicons have also shown modest differences in methylation between dementia and MCI groups. Specifically, 12 differentially methylated cytosines (DMCs) (8 from a total of 22 in BDNF_I2, 2 from a total of 8 in BDNF_IX, and 2 out of a total of 19 in BDNF_IV1) showed differential methylation between two groups when using the sex and age as covariates in the logistic regression F-test and controlling for overdispersion (Supplementary Table S4). However, after correction for multiple testing, only one CpG locus remained statistically significant (positioned in the BDNF_I2 amplicon).

### 2.3. BDNF Gene and Protein Expression Analysis

**Figure 3:**
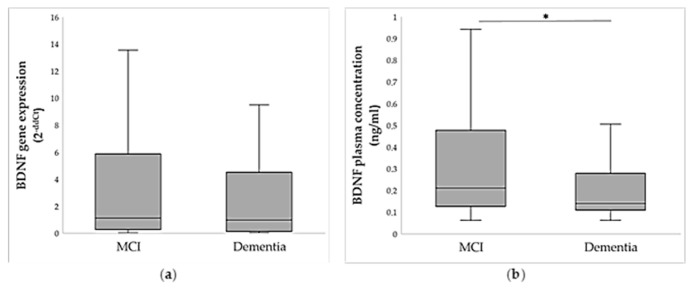
(**a**) Relative *BDNF* gene expression in individuals with MCI (N = 108) and dementia (N = 83), represented as 2^−ddCt^ and (**b**) plasma BDNF protein expression in individuals with MCI (N = 251) and dementia (N = 155). Data are shown as a boxplot diagram. The central box represents the interquartile range, the middle line represents the median, and the vertical line extends from Q1—1.5*IQR and Q3 + 1.5*IQR. * *p* < 0.001, Mann–Whitney U test.

A subset of individuals with MCI and dementia was chosen for relative quantification of *BDNF* gene expression, while BDNF protein expression was analyzed in all participants. As shown in Figure 3a, dementia patients had lower peripheral *BDNF* gene expression in comparison to the MCI group, although this result was not significant (U = 1994.5; *p* = 0.716). Moreover, plasma BDNF protein levels were significantly lower in patients with dementia compared to individuals with MCI (U = 12,366.0; *p* < 0.001; Figure 3b, Supplementary Table S6).

*BDNF* gene expression was significantly positively correlated with BDNF protein concentration (ρ = 0.242; *p* = 0.008). Plasma BDNF protein was especially lower in the severe dementia group (H = 22.2; *p* < 0.001). Specifically, the patients with severe dementia had significantly lower plasma BDNF concentration than persons with mild-to-moderate dementia (*p* = 0.013; post hoc Dunn test) and MCI (*p* < 0.001; post hoc Dunn test), while patients with mild-to-moderate dementia had lower BDNF concentration than individuals with MCI (*p* = 0.006; post hoc Dunn test).

To further control for the effect of sex and age, multiple linear regression was used to determine the effects of age and sex on relative *BDNF* gene and protein expression. There was no significant effect of either of these variables on relative abundance of *BDNF* mRNA in blood or protein concentration in plasma (data available on request). We also tested for the correlation between the percentage of methylation of specific *BDNF* amplicons and *BDNF* gene expression. Methylation of 4 tested amplicons located in the promoters 4 and 1 of the *BDNF* gene showed trend towards negative correlation with *BDNF* gene expression, but none of them was statistically significant (Supplementary Table S5).

### 2.4. Association of BDNF Methylation and Expression with Cognitive Scales

Additionally, we tested the correlation of MMSE and CDT scores with *BDNF* methylation, gene, and protein expression. Multiple linear regression revealed that there was a significant positive correlation between percent of methylation in the BDNF_IV2 region and scores on the CDT scale (*p* = 0.019, Table 3), while the MMSE scale did not show significant association with methylation in the *BDNF* gene. Correlation between *BDNF* gene expression and cognitive scales corrected for the effect of sex and age showed a significant positive correlation between *BDNF* expression and CDT scores (*p* = 0.019, Table 3). The MMSE scale did not correlate significantly with *BDNF* gene expression. On the other hand, BDNF protein levels correlated significantly with the MMSE scores (Table 3), thus confirming the association of plasma BDNF concentration and better cognitive performance.

### 2.5. Association of BDNF Val66Met Polymorphism with BDNF Methylation and Expression

Differences in average methylation levels of *BDNF* amplicons and relative *BDNF* gene expression, as well as plasma concentration, were calculated between carriers of different *BDNF* Val66Met polymorphism genotypes (codominant model: AA vs. GA vs. GG genotype carriers, and dominant model A carriers vs. GG homozygotes). The statistical significance was seen only in association with the BDNF_IX amplicon, located in the IX exon and containing the *BDNF* Val66Met locus as 1 of the total 8 CpG sites, indicating that overall methylation of the BDNF_IX region was associated with *BDNF* Val66Met polymorphism. BDNF_IV1 methylation was marginally higher in *BDNF* Val66Met AA homozygotes compared to heterozygotes (*p* = 0.054), but the dominant model showed non-significantly lower methylation in A carriers compared to GG homozygotes (*p* = 0.824). These discrepancies could be due to a lower number of AA homozygotes or could indicate that the presence of 2 A alleles could be possibly associated with BDNF_IV1 methylation. However, further studies are necessary to confirm these findings. Neither mRNA nor protein levels of BDNF were associated with *BDNF* Val66Met polymorphism (Table 4).

Spearman correlation was performed to further check if the average percent of methylation at the *BDNF* Val66Met site (IX_CpG5, chr11: 27679923) was associated with methylation at *BDNF* loci in the exon IX and other tested *BDNF* amplicons (Table 5).

The highest correlation was observed with adjacent CpG loci (IX_CpG4 and IX_CpG6 with *p* = 5.571 × 10^−13^ and *p* = 4.666E-10 after FDR correction), but also IX_CpG7, IX_CpG8, and IX_CpG1 (Table 5). Moreover, methylation of this site significantly positively correlated with overall methylation in the BDNF_IX amplicon (*p* = 1.459 × 10^−21^) and negatively with BDNF_IV1 (*p* = 0.001), BDNF_IV3 (*p* = 0.002) located in the promoter 4, and with BDNF_I1 (*p* = 0.006), located in the promoter 1 (Table 5). However, it was not associated with *BDNF* gene expression (ρ = 0.163; *p* = 0.132).

### 2.6. Association of BDNF Val66Met Polymorphism with Dementia and Cognitive Scores

Distribution of *BDNF* Val66Met genotypes (codominant model: AA vs. GA vs. GG genotype carriers, and dominant model A carriers vs. GG homozygotes) was evaluated between individuals with dementia and MCI. There was no significant association of *BDNF* Val66Met polymorphism with dementia when individuals were subdivided into dementia and MCI groups, or when dementia patients were further stratified into individuals with mild to moderate dementia and severe dementia (Table 6). Total scores on MMSE and CDT clinical scales used for assessing the cognitive deficits were similar among patients carrying different *BDNF* Val66Met genotypes, indicating a lack of association of *BDNF* Val66Met polymorphism and cognitive decline in individuals with MCI and dementia (Table 6).

## 3. Discussion

This study investigated differences in the distribution of *BDNF* Val66Met (rs6265) variants, as well as in peripheral *BDNF* DNA methylation, gene expression, and protein levels, between persons with MCI and dementia. It also explored their associations with cognitive performances assessed by MMSE and CDT scales.

Differences in DNA methylation of 6 chosen regions in *BDNF* promoters and exon IX were not statistically significant between individuals with dementia and MCI. However, hierarchical logistic regression, adjusted for age and sex, demonstrated that lower BDNF_IV1 methylation had significant predictive value for dementia. Previous research indicated that *BDNF* promoter IV methylation is highly responsive to environmental stimuli and correlates with *BDNF* gene expression [18]. Our previous study [17] demonstrated borderline significantly lower methylation levels in the *BDNF*_IV2 region, as well as reduced peripheral *BDNF* gene expression among severe AD patients compared to persons with MCI. On the other hand, results of this study demonstrated that patients with mild-to-moderate dementia had lower *BDNF*_IV2 methylation than MCI, whereas patients with severe dementia had higher levels of DNA methylation in this region compared to other groups. Our findings could indicate non-linear changes in *BDNF* methylation because of compensatory mechanisms. Discrepancies with our previous findings could be due to the differences in diagnosis of enrolled individuals or severity of cognitive symptoms.

This present study also observed the trend for lower expression of peripheral *BDNF* mRNA and significantly lower BDNF plasma levels in patients with dementia, especially severe dementia. *BDNF* gene and protein expression can also be influenced by non-coding transcripts or other post-transcriptional modifications regulating BDNF protein levels [19]. Expression of the *BDNF* gene does not necessarily reflect the rate of total BDNF synthesis [20], since human platelets are the primary source of serum BDNF protein but not mRNA [21,22]. Moreover, it is not known if peripheral cells reflect the pattern of *BDNF* expression in the brain; however, it is hypothesized that BDNF could pass the blood–brain barrier, particularly under pathological conditions [23]. Nevertheless, in our study, *BDNF* gene expression was positively correlated with plasma BDNF protein concentration and CDT scores, whereas BDNF protein levels were positively correlated with MMSE scores. In line with our findings, some previous studies [24,25,26,27], but not all [28,29,30,31], reported lower BDNF levels in AD patients. Discrepancies may be due to factors such as age, gender, disease stage, or methodology for BDNF determination [26,32,33]. Some studies indicated transiently elevated BDNF levels in early stages of dementia, probably due to compensatory or repair mechanisms [32,34,35,36].

None of the *BDNF* amplicons investigated in our study showed methylation correlation with *BDNF* gene expression. However, there was a significant positive correlation of percent of methylation in the BDNF_IV2 region with scores on the CDT scale. Although amplicons within promotor BDNF_I did not show different methylation between investigated groups, most of the individual CpG loci that showed significantly different DNA methylation between individuals with dementia and MCI were in the BDNF_I2 amplicon of *BDNF* promoter I. Previous studies have also linked methylation alternations in *BDNF* promoters I and IV with cognitive deficits related to visuospatial abilities, memory [37], and neuropsychiatric symptoms of AD [11,12,38]. *BDNF* promotor hypermethylation in the postmortem hippocampus and frontal cortex of AD patients has been reported, supporting observed reductions in *BDNF* mRNA or protein levels in these brain areas [9,39,40].

The *BDNF* rs6265 (G > A, Val66Met) functional polymorphism located in the exon IX of the human pro-BDNF sequence influences intracellular trafficking, reduces activity-dependent secretion of BDNF, and is associated with memory-related behavior and brain activity in animal models and humans [14]. The Met (A) allele was mostly reported as a risk factor for various neuropsychiatric disorders, including AD [41,42], while results regarding cognitive performance are more complex [41,43]. Although the Met allele is usually associated with poorer cognitive functions such as short-term visual memory and attention in PTSD [44] and smaller hippocampal volume in AD [41,45], some studies suggest that Met allele carriers with depression have better performance in executive functions but worse memory performance compared to Val/Val carriers [43]. This indicated that *BDNF* Val66Met polymorphism might have differential effects on cognitive functions in the presence of psychopathology and could modulate the effect of age and severity of psychiatric disorders on cognitive performance, possibly through *BDNF* methylation [16,41,46].

In our study we observed no significant association of *BDNF* Val66Met polymorphism with MMSE and CDT scores, development of dementia, peripheral *BDNF* gene or protein expression, or methylation of regions within *BDNF* promoters I and IV. However, it was significantly associated with average methylation of the BDNF_IX amplicon in the exon IX coding region. This region includes *BDNF* Val66Met polymorphism as a potential methylation site depending on the presence of the G or A allele, thus forming a CpG or CpA locus [20]. As expected, methylation of BDNF_IX was higher in Val/Val (GG) carriers than in Val/Met (GA) or Met/Met (AA) carriers, since most cytosine methylation occurs at CpG sites, although it is also possible at CpA sites [47].

In addition, methylation at the *BDNF* Val66Met polymorphism site was positively correlated with overall methylation of the BDNF_IX region and 5 adjacent CpG loci, especially BDNF_IX _CpG4 and BDNF_IX _CpG6, surrounding BDNF_IX _CpG5 (Val66Met) site. This is in line with the study which reported the association of *BDNF* Val66Met polymorphism and DNA methylation at surrounding CpG sites in frontal cortex brain tissue [15] and the general theory that nearby CpG sites are commonly methylated together, possibly due to the preference of DNA methyltransferases to methylate CpG pairs at a particular distance range or due to the influence of neighboring CpG sites in the recruitment of DNA methylase and/or demethylase enzymes [48].

On the other hand, DNA methylation at the BDNF_IX_CpG5 (Val66Met) site negatively correlated with methylation in BDNF_IV1, BDNF_IV3, and BDNF_I1 regions, located in *BDNF* promoters IV and I. This is not surprising if we consider the different effects of CpG hypermethylation depending on whether it occurs in the gene body (increase in gene expression), intragenic region (influence on gene splicing), or promoter or enhancer regions (blocking the binding of transcriptional factors and transcriptional silencing) [18,47]. Although in our study the *BDNF* Val66Met polymorphism did not show association with dementia or neurocognitive symptoms, it might have influenced dementia via differential methylation of *BDNF* promotor regions, which showed modest association with severe dementia and visuospatial cognitive impairments.

## 4. Materials and Methods

### 4.1. Participants

The study included a total of 406 (177 female) unrelated Caucasian participants, of which 251 had mild cognitive impairment (MCI) (mean age 67.34 ± 5.63, ranging from 56 to 89 years) and 155 persons with dementia (mean age 69.88 ± 6.30, ranging from 58 to 89 years), recruited from the University Psychiatric Hospital Vrapce, Zagreb, Croatia. Diagnosis of dementia and MCI was based on DSM-5 criteria [49] and cognitive abilities were assessed using neurocognitive scales, the Mini-Mental State Examination (MMSE) [50], and the Clock Drawing Test (CDT) [51]. Additionally, we have subdivided dementia patients according to the severity of symptoms. However, since the group of patients with moderate dementia was small (~10 patients), we have subdivided dementia patients into two groups: mild-to-moderate dementia and severe dementia, and compared them with the MCI group.

Individuals diagnosed with terminal illnesses or serious genetic and somatic diseases, as well as other psychiatric or neurological disorders, were excluded from the study. Participants were included in the study after signing the informed written consent and before they were treated with anti-dementia drugs, if necessary. The study was carried out in accordance with the Declaration of Helsinki and approved by the Ethics Committees of University Psychiatric Hospital Vrapce and Ruder Boskovic Institute, Zagreb, Croatia.

### 4.2. Blood Collection

Whole blood samples (8.5 mL) were collected at 8 a.m., after an overnight fast, in BD Vacutainer™ tubes with 1.5 mL of acid citrate dextrose anticoagulant. Sampling was performed during routine laboratory visits. Blood samples were stored at 4 °C and delivered to the Ruder Boskovic Institute. Upon arrival, blood samples were immediately processed, and plasma was separated from the whole blood by centrifugation (3 min at 1100× *g*, followed by 15 min at 5030× *g*) and stored in aliquots at −20 °C until further analysis. To maintain sample integrity, repeated freeze–thaw cycles were avoided. The remaining blood fraction was used for the isolation of total DNA and RNA.

### 4.3. Methylation Analysis

#### 4.3.1. DNA Isolation and Bisulfite Conversion

Extraction of genomic DNA was carried out using the PureLink™ Genomic DNA Mini Kit (Invitrogen, Carlsbad, CA, USA) according to the manufacturer’s protocol. DNA samples were stored at −20 °C until further analysis. Bisulfite conversion was performed on 800 ng of DNA using an EpiTect Fast Bisulfite Kit (Qiagen, Venlo, The Netherlands) following the manufacturer’s protocol. After measuring the concentration and quality of DNA and the volume of unconverted DNA, the elution volume was adjusted so that the final concentration of bisulfite-converted DNA (bsDNA) was 20 ng/µL.

#### 4.3.2. Primer Design

CpG island (CGI) sequences of the *BDNF* gene with additional 500-base-pair flanking regions upstream and downstream of the CGI were obtained from the UCSC genome browser (Homo sapiens version hg19, http://genome.ucsc.edu/ (accessed on 15 July 2023.)) [52]. For primer design, the Methyl Primer Express (v1.0, https://resource.thermofisher.com/page/WE28396_2/ (accessed on 15 July 2023)) was used. Primer pairs were designed to amplify approximately 300-base-pair-long regions within promotor regions of the first (I) and fourth exon (IV) of the *BDNF* gene, as well as the coding region of the exon IX containing the *BDNF* Val66Met (rs6265) polymorphism site. Specificity and properties of designed primers were determined using IDT oligo analyzer (https://eu.idtdna.com/pages/tools/oligoanalyzer (accessed on 30 July 2023)). To DNA sequence-specific primers, Illumina adapter overhang sequences were added to the 5′ end. Amplicon positions and lengths and number of CpGs are listed in Table 7, whereas primer pairs are listed in Supplementary Table S1.

#### 4.3.3. Amplicon Generation

The amplicon library was prepared according to Illumina 16S protocol [53] as previously described [17]. Briefly, target sequence amplification was conducted in two PCR rounds. In the first round, target regions were amplified using primer pairs listed in Supplementary Table S1. PCR reactions were carried out in 25 µL total volume and contained 12.5 µL of KAPA HiFi HotStart Uracil+ ReadyMix (Roche, KAPA Biosystems Ltd., Cape Town, South Africa), 1 µM of each primer, and 20 ng of DNA. The thermal cycling protocol included initial activation at 95 °C for 5 min, followed by 35 cycles of denaturation at 98 °C for 30 s, primer-specific annealing for 15 s (Supplementary Table S1), and extension at 72 °C for 15 s. A final extension step at 72 °C for 1 min was followed by a hold at 4 °C. After the first round of amplification, fragments were visualized via 2% agarose gel electrophoresis to determine the generation of amplicons of appropriate lengths (approximately 300 bp). Cleanup of shorter, unspecific fragments was performed using AMPure XP beads (Beckman Coulter, Brea, CA, USA). Subsequently, all amplicons from each subject were pooled in equimolar amounts, with concentrations measured using the Quant-iT PicoGreen dsDNA assay (Thermo Scientific, Life Technologies, Waltham, MA, USA). The second round of PCR, in which Nextera XT v2 index set A and set D primers (Illumina, San Diego, CA, USA) were used, was performed on pooled samples in order to incorporate subject-specific indices for multiplexing. PCR reactions were carried out in a total volume of 50 μL, containing 25 μL of KAPA HiFi HotStart Uracil+ ReadyMix (Roche, Basel, Switzerland), Nextera XT v2 primers, and 4 ng of equimolar amplicon pool. PCR conditions included initial activation at 98 °C for 45 s, 10 cycles of 15 s at 98 °C, 30 s at 55 °C, and 30 s at 72 °C, with final extension at 72 °C for 1 min. Amplification products were visualized on a 2% agarose gel to confirm the presence of correctly sized amplicons.

#### 4.3.4. Library Preparation and Next-Generation Sequencing

After the second round of PCR amplification and size selection (AMPure XP paramagnetic beads, Beckman Coulter, Brea, CA, USA), DNA concentration in libraries was measured using PicoGreen dsDNA quantitation reagent (Thermo Fisher, Waltham, MA, USA). Individual libraries were equimolarly pooled into a final library with a 10 nM molar concentration and diluted and denatured according to Illumina MiniSeq System Denature and Dilute Libraries Guide recommendations. The library was sequenced using an Illumina MiniSeq sequencer, MiniSeq Mid Output Kit (300 cycles), and 150 bp paired-end reads.

### 4.4. Gene Expression

#### 4.4.1. RNA Isolation and Reverse Transcription

RNA extraction from blood was performed using the NucleoSpin RNA Blood kit (Macherey-Nagel, Düren, Germany) according to the manufacturer’s instructions. RNA was eluted in 60 μL of RNase-free water. Quantity and quality of RNA samples were determined using a NanoPhotometer^®^ C40 (Implen, München, Germany). Total RNA was transcribed into complementary DNA (cDNA) using a Verso cDNA Synthesis Kit (Thermo Fisher Scientific, Life Technologies, Waltham, MA, USA) in a total of 20 μL of reaction according to the manufacturer’s protocols. cDNA samples were stored at −80 °C.

#### 4.4.2. Real-Time PCR and Comparative Ct (ΔΔCt) Method

*BDNF* gene expression from blood of individuals with MCI and dementia was determined using the ABI 7300 Real-Time PCR System (Applied Biosystems, Foster City, CA, USA) using TaqMan Gene Expression Assays—Hs02718934_s1 for *BDNF* and Hs00266705_g1 for glyceraldehyde-3-phosphate dehydrogenase (*GADPH*) gene (Applied Biosystems, Foster City, CA, USA), which was used as a housekeeping gene for normalization. Amplification reactions were performed in triplicate. After initial denaturation at 95 °C for 10 min, 43 cycles of 95 °C for 15 s and 60 °C for 1 min followed. Comparative Ct (ΔΔCt) method was used for determining relative *BDNF* expression in blood, which was represented as a 2^−ΔΔCt^ value. The average Ct of all MCI samples was used as a reference. Cycle threshold (Ct) values were determined using SDS software v1.3 (Applied Biosystems, Foster City, CA, USA). Relative gene expression was represented as fold-change (2^−ΔΔCt^) compared to average Ct of all MCI samples which were present in every plate.

### 4.5. Determination of BDNF Plasma Concentration

BDNF plasma concentrations were quantified using a commercial sandwich enzyme-linked immunosorbent assay (ELISA) kit (ELK5404), following the manufacturer’s protocols (ELK Biotechnology, Inc., Denver, CO, USA). All plasma samples were analyzed in duplicate. Plasma samples and appropriately diluted standards were added to 96-well plates pre-coated with monoclonal antibodies specific to human BDNF, incubated for 80 min at 37 °C, and then washed 3 times with washing buffer. A biotinylated antibody was added, and plates were incubated for 50–60 min at 37 °C and then washed. Streptavidin-conjugated horseradish peroxidase (HRP) was subsequently added, followed by another 50–60 min incubation at 37 °C. After five washes, a substrate solution was added, and plates were incubated for 20 min at 37 °C in the dark. The enzymatic reaction was terminated by adding Stop Solution. Absorbance was measured at 450 nm using a microplate reader. Sample concentrations were calculated based on the corresponding standard curve.

### 4.6. Genotyping

Genotyping of *BDNF* rs6265 (Val66Met) polymorphism was described in detail before [54]. Genotyping was performed by a real-time PCR method using Applied Biosystems^®^ 7300 Real-Time PCR System apparatus and TaqMan^®^ Genotyping Assay C_11592758_10 (Applied Biosystems, Foster City, CA, USA) according to the manufacturer’s protocol. Aside from the codominant model, which included all three genotypes, due to the low minor allele frequency of *BDNF* Val66Met polymorphism, we have also evaluated the dominant model (AA+GA carriers vs. GG).

### 4.7. Biostatistical and Statistical Analysis

Raw sequencing reads were assessed for quality testing using FastQC [55], whereas Trim Galore [56] was used to trim adapter sequences and bases of poor quality. Trimmed sequences were then aligned to the reference UCSC genome browser (Homo sapiens Human Genome version 19, hg19) using Bismark [57]. Aligned reads were further analyzed using the R Statistics 4.2.2. Samples with coverage reads < 5 on most of the sites were excluded from analysis. Differentially methylated cytosines (DMCs) were identified using the methylKit package [58] and built-in logistic regression F test, with mean normalized overdispersion, age, and sex variables used as covariates (to separate their influence from dementia effect), as well as with Benjamini–Hochberg adjustment for multiple testing. Average percent of methylation was calculated for 6 tested *BDNF* amplicons, based on individual CpG methylation values. Normality of CpG value distribution was assessed using the Kolmogorov–Smirnov normality test. Due to deviation of normality, nonparametric statistical tests were used as follows: the Mann–Whitney U test and Kruskal–Wallis test for calculation of differences in average methylation between 2 or more than 2 groups, respectively; Spearman correlation for comparing average methylation with gene and protein expression or severity of dementia symptoms; and the chi-square test to check for different distribution of *BDNF* Val66Met genotypes between diagnostic groups. Hierarchical logistic regression was used to test the effect of age and sex as predictors of dementia, in addition to tested BDNF amplicons. Two models have been constructed: a base model with age and sex as only predictors and the test model with added average percent of methylation of all tested BDNF amplicons. Outlier analysis was performed using Tukey’s method, which uses interquartile range (IQR) based on the formula: low outliers = Q1—1.5*IQR and high outliers = Q3 + 1.5*IQR, where Q1 is the 1st quartile and Q3 is the 3rd quartile. Since we wanted to account for biological variability, we did not exclude all outliers, but only the extreme values, which were identified as data points beyond 3 times the IQR from the 1st or 3rd quartile. Multiple linear regression was used to determine the effects of age and sex on MMSE and CDT scores, and standardized residuals were used to test the correlation of MMSE and CDT scores with *BDNF* methylation and expression. *p*-values < 0.05 were considered statistically significant.

## 5. Conclusions

In blood samples of individuals with MCI and dementia, we have investigated DNA methylation of six regions in the *BDNF* gene, *BDNF* gene and protein expression, and association of *BDNF* Val66Met polymorphism with dementia and neurocognitive changes assessed by MMSE and CDT scales. Out of six tested *BDNF* amplicons, two are located within the exon I promotor, three in promotor IV, and one in the exon IX coding region, which includes *BDNF* Val66Met polymorphism as a differential methylation site based on the presence of the G or A allele.

Two amplicons in promoter IV showed modest but significant association with dementia and neurocognitive symptoms. Specifically, lower BDNF_IV1 methylation demonstrated predictive value for dementia. Moreover, patients with mild-to-moderate dementia had lower levels of BDNF_IV2 methylation, whereas patients with severe dementia had higher levels in comparison to the MCI group. BDNF_IV2 methylation also positively correlated with CDT scores. In addition, individual CpG sites in BDNF_I2 in promotor I showed significantly different DNA methylation between individuals with dementia and MCI. An insignificant decline in *BDNF* mRNA levels in dementia patients positively correlated with significantly lower BDNF plasma levels, especially pronounced in severe dementia patients. In addition to a significant positive correlation between *BDNF* mRNA levels and CDT scores, plasma BDNF protein levels positively correlated with MMSE scores. *BDNF* Val66Met polymorphism was associated with methylation of the BDNF_IX amplicon, but not with methylation in *BDNF* promoters I and IV, peripheral BDNF gene and protein expression, neurocognitive symptoms detected with MMSE and CDT scales, or with dementia. Methylation at the *BDNF* Val66Met site was positively correlated with overall BDNF_IX methylation and methylation at 5 adjacent CpG sites in exon IX but negatively correlated with methylation of BDNF_IV1, BDNF_IV3, and BDNF_I1 amplicons in *BDNF* promoters I and IV, suggesting the possibility of an indirect effect of *BDNF* Val66Met polymorphism on dementia and cognition via methylation of *BDNF* promotor regions.

Strengths of this study are the relatively large sample size and use of two different scales, MMSE and CDT, for evaluation of cognitive functions. Moreover, BDNF association with dementia and cognitive functioning has been assessed on three different levels (methylation, expression, and genotype), while correcting for age and sex of participants. However, besides age and sex, additional factors that may affect BDNF levels, such as body mass index, nicotine and alcohol consumption, physical exercise, dietary patterns, stress, as well as use of psychotropic drugs, should be carefully considered. On the other hand, limitations of this study include enrollment of a mixed cohort of patients with different types of dementia, such as AD, vascular dementia, frontotemporal dementia, etc. Therefore, in further studies, MRI imaging data could be used to define dementia patients in more detail, since cognitive deficits may significantly vary between various dementia types due to different brain areas which are affected. Nevertheless, we assume that most dementia patients enrolled in our study have AD, since this neurodegenerative disorder accounts for 60–80% of all dementia cases. Future studies should also enroll more patients with moderate dementia, whose number was relatively small in this study. Another limitation of our study is the lack of the age-matched control group of participants without cognitive impairment, which comparison with MCI and dementia patients might add to the study’s value.

Despite these limitations, our findings demonstrate complex interactions between peripheral *BDNF* methylation status, gene and protein levels, *BDNF* Val66Met genotype, and cognitive impairments, which have been so far hard to interpret. Therefore, further studies should evaluate the translational potential of observed BDNF alternations as blood-based biomarkers for clinical diagnosis and monitoring of dementia, as well as the possible usefulness of BDNF replacement in cognitive disorders.

## Figures and Tables

**Table 1:** Demographic and clinical data of individuals with MCI and dementia.

Demographic Data	MCI	Dementia	Statistics *
N (Male)	153 (61.0%)	76 (49.0%)	χ^2^ = 5.541; ***p* = 0.011**
N (Female)	98 (39.0%)	79 (51.0%)	
Age	68 (64; 71)	70 (65; 72)	U = 15,622.5; ***p* = 0.001**
**Neurocognitive clinical scales**			
MMSE	27 (26; 29)	22 (20; 23)	U = 0.0; ***p* < 0.001**
CDT	4 (4; 5)	3 (2; 5)	U = 11,845.0; ***p* < 0.001**

Data are shown as median (25th; 75th percentile). * χ^2^-test and Mann–Whitney U test; *p*-values in bold are statistically significant. CDT—Clock Drawing Test; MCI—mild cognitive impairment; MMSE—Mini-Mental State Examination.

**Table 2:** Hierarchical logistic regression testing the effects of age, sex, and methylation of 6 *BDNF* amplicons as predictors of dementia.

	Base Model			Test Model		
Amplicon	β	95% CI	*p*-Value	β	95% CI	*p*-Value
Age	1.066	1.012–1.123	**0.016**	1.074	1.014–1.139	**0.016**
Sex (male)	0.464	0.238–0.903	**0.024**	0.413	0.202–0.841	**0.015**
BDNF_IX				0.931	0.849–1.022	0.133
BDNF_IV1				0.512	0.309–0.847	**0.009**
BDNF_IV2				0.802	0.313–2.056	0.646
BDNF_IV3				1.527	0.822–2.835	0.180
BDNF_I1				1.508	0.914–2.486	0.108
BDNF_I2				0.950	0.737–1.223	0.689
**Model** **statistics**	χ^2^ = 10.784; *p* = **0.005** −2 Log Likelihood = 202.4. R^2^ = 0.090			χ^2^ = 25.282; *p* = **0.001** −2 Log Likelihood = 187.7; R^2^ = 0.201		

*p*-values in bold are statistically significant. BDNF—Brain-Derived Neurotrophic Factor.

**Table 3:** Correlation of methylation of *BDNF* amplicons, *BDNF* gene and protein expression with MMSE and CDT scales, corrected for the effect of sex and age.

Amplicon	MMSE	CDT
BDNF_IX	ρ = 0.072; *p* = 0.313	ρ = 0.068; *p* = 0.341
BDNF_IV1	ρ = 0.070; *p* = 0.330	ρ = −0.015; *p* = 0.829
BDNF_IV2	ρ = −0.027; *p* = 0.708	ρ = 0.168; ***p* = 0.019**
BDNF_IV3	ρ = −0.006; *p* = 0.936	ρ = −0.066; *p* = 0.372
BDNF_I1	ρ = −0.014; *p* = 0.843	ρ = 0.073; *p* = 0.312
BDNF_I2	ρ = −0.018; *p* = 0.815	ρ = 0.031; *p* = 0.679
*BDNF* gene expression	ρ = 0.095; *p* = 0.284	ρ = 0.205; ***p* = 0.019**
BDNF protein expression	ρ = 0.211; ***p* < 0.001**	ρ = 0.061; *p* = 0.243

Relative BDNF expression was calculated as 2^−ddCt^. Spearman correlation: *p*-values in bold are statistically significant. BDNF—Brain-Derived Neurotrophic Factor; CDT—Clock Drawing Test; MMSE—Mini-Mental State Examination.

**Table 4:** Association of *BDNF* Val66Met polymorphism with average percent of methylation of *BDNF* amplicons and *BDNF* gene and protein expression.

	*BDNF* Val66Met Genotypes				*BDNF* Val66Met Carriers		
	AA	GA	GG	Statistics *	A carriers	GG	Statistics **
BDNF_IX	82.263 (77.201; 89.935)	85.035 (82.749; 86.069)	89.115 (87.026; 90.782)	H = 60.29; ***p* < 0.001**	85.035 (82.371; 86.208)	89.115 (87.026; 90.782)	U = 1400.0; ***p* < 0.001**
BDNF_IV1	3.279 (2.888; 3.793)	1.683 (1.240; 2.314)	1.811 (1.351; 2.273)	H = 5.83; *p* = 0.054	1.694 (1.246; 2.591)	1.811 (1.351; 2.273)	U = 4239.0; *p* = 0.824
BDNF_IV2	0.765 (0.698; 1.084)	0.611 (0.438; 1.089)	0.644 (0.411; 0.873)	H = 1.20; *p* = 0.550	0.647 (0.444; 1.087)	0.644 (0.411; 0.873)	U = 3896.0; *p* = 0.423
BDNF_IV3	1.630 (1.386; 2.066)	1.731 (1.404; 2.047)	1.558 (1.249; 1.972)	H = 1.88; *p* = 0.391	1.724 (1.403; 2.048)	1.558 (1.249; 1.972)	U = 3456.0; *p* = 0.172
BDNF_I1	2.179 (1.685; 3.322)	1.426 (1.017; 2.114)	1.451 (0.947; 2.113)	H = 3.21; *p* = 0.201	1.493 (1.058; 2.248)	1.451 (0.947; 2.113)	U = 3998.5; *p* = 0.601
BDNF_I2	4.954 (1.878; 5.339)	2.662 (2.186; 3.538)	2.524 (1.952; 3.436)	H = 3.67; *p* = 0.160	2.684 (2.112; 3.786)	2.524 (1.952; 3.436)	U = 3157.0; *p* = 0.133
*BDNF* gene expression	-	3.693 (0.180; 6.720)	0.795 (0.263; 4.611)	-	3.693 (0.180; 6.720)	0.795 (0.263; 4.611)	U = 1405.0; *p* = 0.296
BDNF protein expression	0.222 (0.145; 0.708)	0.191 (0.127; 0.448)	0.164 (0.113; 0.412)	H = 3.491; *p* = 0.175	0.195 (0.127; 0.451)	0.166 (0.113; 0.408)	U = 14428.0; *p* = 0.099

Data are shown as median (25th; 75th percentile). Relative *BDNF* gene expression was calculated as 2^−ddCt^. * Kruskal–Wallis test; ** Mann–Whitney U test; *p*-values in bold are statistically significant. BDNF—Brain-Derived Neurotrophic Factor.

**Table 5:** Correlation in methylation of the *BDNF* CpG locus corresponding to the *BDNF* Val66Met polymorphism (chr11: 27679923) with CpG loci in exon IX and other tested *BDNF* amplicons.

Amplicon	Location (hg19)	Statistics *
IX_CpG1	chr11: 27,679,840	*p* = 0.260; ***p* = 0.001**
IX_CpG2	chr11: 27,679,854	*p* = 0.083; *p* = 0.286
IX_CpG3	chr11: 27,679,880	*p* = 0.076; *p* = 0.289
IX_CpG4	chr11: 27,679,917	*p* = 0.503; ***p* = 5.571 × 10^−13^**
IX_CpG5 (rs6265)	chr11: 27,679,923	-
IX_CpG6	chr11: 27,679,977	*p* = 0.437; ***p* = 4.666 × 10^−10^**
IX_CpG7	chr11: 27,680,000	*p* = 0.256; ***p* = 0.001**
IX_CpG8	chr11: 27,680,033	*p* = 0.233; ***p* = 0.001**
BDNF_IX	chr11: 27,679,766–27,680,064	*p* = 0.621; ***p* = 1.459 × 10^−21^**
BDNF_IV1	chr11: 27,721,638–27,721,854	*p* = −0.259; ***p* = 0.001**
BDNF_IV2	chr11: 27,722,209–27,722,487	*p* = −0.091; *p* = 0.296
BDNF_IV3	chr11: 27,723,104–27,723,373	*p* = −0.245; ***p* = 0.002**
BDNF_I1	chr11: 27,743,454–27,743,762	*p* = −0.210; ***p* = 0.006**
BDNF_I2	chr11: 27,744,260–27,744,605	*p* = 0.027; *p* = 0.844

* Spearman correlation: *p*-values in bold are statistically significant after FDR correction. BDNF—Brain-Derived Neurotrophic Factor.

**Table 6:** Association of *BDNF* Val66Met polymorphism with dementia and related cognitive impairment scores.

		*BDNF* Val66Met Genotypes			*BDNF* Val66Met Carriers			
		AA	GA	GG	Statistics *	A Carriers	GG	Statistics **
Diagnosis	MCI	10 (76.9%)	74 (60.7%)	149 (62.1%)	χ^2^ = 1.322 *p* = 0.516	84 (62.2%)	149 (62.1%)	χ^2^ = 0.001 *p* = 0.979
	Dementia	3 (23.1%)	48 (39.3%)	91 (37.9%)		51 (37.8%)	91 (37.9%)	
Severity of cognitive decline	MCI	10 (76.9%)	74 (60.7%)	149 (62.1%)	χ^2^ = 2.800 *p* = 0.592	84 (62.2%)	149 (62.1%)	χ^2^ = 1.472 *p* = 0.479
	Mild to moderate	2 (15.4%)	35 (28.7%)	74 (30.8%)		37 (27.4%)	74 (30.8%)	
	Severe	1 (7.7%)	13 (10.7%)	17 (7.1%)		14 (10.4%)	17 (7.1%)	
Scores on cognitive scales	MMSE	26 (25; 27)	26 (22; 27)	25 (23; 27)	H = 0.02; *p* = 0.990	26 (23; 27)	25 (23; 27)	U = 16192.5; *p* = 0.994
	SAT	4 (3; 5)	4 (3; 5)	4 (3; 5)	H = 1.37; *p* = 0.505	4 (3; 5)	4 (3; 5)	U = 15023.0; *p* = 0.243

Data are shown as total number (percentage) or as median (25th; 75th percentile). * χ^2^-test and Kruskal–Wallis test; ** χ^2^-test and Mann–Whitney U test. BDNF—Brain-Derived Neurotrophic Factor; CDT—Clock Drawing Test; MCI—Mild Cognitive Impairment; MMSE—Mini-Mental State Examination.

**Table 7:** List of tested amplicons with their locations based on hg19.

Amplicon	Chromosome	Start Position	End Position	Location	Strand, Length	Number of CpGs
BDNF_IX	chr11	27679766	27680064	Exon IX (coding)	(−), 299	8
BDNF_IV1	chr11	27721638	27721854	Promoter of the exon IV	(−), 217	19
BDNF_IV2	chr11	27722209	27722487	Promoter of the exon IV	(−), 279	23
BDNF_IV3	chr11	27723104	27723373	Promoter of the exon IV	(−), 270	15
BDNF_I1	chr11	27743454	27743762	Promoter of the exon I	(−), 309	20
BDNF_I2	chr11	27744260	27744605	Promoter of the exon I	(−), 346	22

## Data Availability

Original data are available from the corresponding author upon reasonable request.

## Supplementary Materials

The following supporting information can be downloaded at: https://www.mdpi.com/article/10.3390/ijms26188987/s1.

## Abbreviations

The following abbreviations are used in this manuscript:

BDNF	Brain-Derived Neurotrophic Factor
CDT	Clock Drawing Test
MCI	Mild Cognitive Impairment
MMSE	Mini-Mental State Examination
