# Supplementary material for: BDNF Val66Met Genotype, DNA Methylation, mRNA, and Protein Levels as Potential Blood-Based Biomarkers for Dementia and Cognitive Decline
Source: Int J Mol Sci. 2025 Sep 15;26(18):8987. doi: 10.3390/ijms26188987 (PMC12470092; doi:10.3390/ijms26188987)
Supplement: Supplementary file 1 [file ijms-26-08987-s001.zip › ijms-3828922-supplementary.pdf]

Supplementary Material

Supplementary Table S1. *BDNF* amplicons primer sequences with Illumina adapter overhang sequences at 5′ end of forward and reverse primers and annealing temperatures

Amplicon	Primer pairs	Annealing temperature
BDNF_IX	Fw: TCGTCGGCAGCGTCAGATGTGTATAAGAGACAG**GAAGGTTGTTTTTATGAAAGAAGTAA**<br>Rev: GTCTCGTGGGCTCGGAGATGTGTATAAGAGACAG**AATTTTTATATTCCTCCAACAAAAA**	50.2°C
BDNF_IV1	Fw: TCGTCGGCAGCGTCAGATGTGTATAAGAGACAG**GGTTTTAATGAGATATTTAT**<br>Rev: GTCTCGTGGGCTCGGAGATGTGTATAAGAGACAG**AAAATCCCCCAATCAACTCTCT**	58.0°C
BDNF_IV2	Fw: TCGTCGGCAGCGTCAGATGTGTATAAGAGACAG**NNTTTTTTAGAGAATTTGGGTGT**<br>Rev: GTCTCGTGGGCTCGGAGATGTGTATAAGAGACAG**NNAAACCTATCCTCACCTCCT**	56.7°C
BDNF_IV3	Fw: TCGTCGGCAGCGTCAGATGTGTATAAGAGACAG**GGGTTGGAAGTGAAAATATTTGTAA**<br>Rev: GTCTCGTGGGCTCGGAGATGTGTATAAGAGACAG**CAAAAAAATTTCATACTAATTC**	50.2°C
BDNF_I1	Fw: TCGTCGGCAGCGTCAGATGTGTATAAGAGACAG**TTTTTAGTTATGATGGGGGAGG**<br>Rev: GTCTCGTGGGCTCGGAGATGTGTATAAGAGACAG**CAAATCACACCTAAAACTCC**	58.3°C
BDNF_I2	Fw: TCGTCGGCAGCGTCAGATGTGTATAAGAGACAG**TGAGGGTAGGTAAAGGGTAGT**<br>Rev: GTCTCGTGGGCTCGGAGATGTGTATAAGAGACA**ACTCTCCCAAAAAACCCTAC**	55.3°C

Supplementary Table S2. Average % of methylation in individuals with MCI and dementia

Amplicon	MCI (% methylation)	Dementia (% methylation)	Mean Difference*	Statistics**
BDNF_IX	87.89 (85.10; 90.46)	87.01 (84.86; 90.10)	0.509	U=4380.0; p=0.308
BDNF_IV1	1.87 (1.24; 2.46)	1.66 (1.34; 2.30)	0.168	U=4573.0; p=0.543
BDNF_IV2	0.65 (0.42; 0.84)	0.64 (0.42; 0.93)	-0.037	U=4529.0; p=0.841
BDNF_IV3	1.59 (1.24; 1.94)	1.63 (1.32; 2.05)	-0.208	U=3949.0; p=0.270
BDNF_I1	1.38 (0.89; 2.09)	1.47 (1.02; 2.30)	-0.143	U=4290.0; p=0.394
BDNF_I2	2.67 (1.95; 3.67)	2.61 (1.99; 3.51)	0.009	U=3924.0; p=0.935

Data are shown as median (25th; 75th percentile). *Difference between groups in average % of methylation per amplicon. **Mann-Whitney U test. BDNF - Brain-Derived Neurotrophic Factor; MCI - Mild Cognitive Impairment

Supplementary Table S3. Average % of methylation in individuals with MCI, mild to moderate and severe dementia

Amplicon	MCI (% methylation)	Mild/moderate dementia (% methylation)	Severe dementia (% methylation)	Statistics*
BDNF_IX	87.89 (85.10; 90.46)	87.45 (84.78; 89.63)	86.35 (85.12; 90.70)	H=1.48; p=0.476
BDNF_IV1	1.87 (1.24; 2.46)	1.62 (1.32; 2.29)	1.71 (1.39; 2.31)	H=0.62; p=0.733
BDNF_IV2	0.65 (0.42; 0.84)	0.57 (0.39; 0.90)	0.82 (0.51; 1.17)	H=6.18; **p=0.045**
BDNF_IV3	1.59 (1.24; 1.94)	1.72 (1.32; 2.05)	1.59 (1.25; 2.07)	H=1.23; p=0.541
BDNF_I1	1.38 (0.89; 2.09)	1.37 (1.01; 2.08)	1.62 (1.12; 2.69)	H=2.28; p=0.320
BDNF_I2	2.67 (1.95; 3.67)	2.57 (1.93; 3.44)	2.79 (2.19; 3.54)	H=0.58; p=0.749

Data are shown as median (25th; 75th percentile). * Kruskal-Wallis test; p-values in bold are statistically significant. BDNF - Brain-Derived Neurotrophic Factor; MCI - Mild Cognitive Impairment

Supplementary Table S4. All identified differentially methylated cytosines in six *BDNF* amplicons and their % difference in methylation between individuals with MCI and dementia.

Chromosome	Amplicon	Position (hg19)	strand	p-value	p-adj	Methylation difference
chr11	BDNF_IX	27679840	-	0.757	0.951	-0.080
chr11	BDNF_IX	27679854	-	0.205	0.633	0.184
chr11	BDNF_IX	27679880	-	0.101	0.545	1.599
chr11	BDNF_IX	27679917	-	**0.038**	0.414	-6.348
chr11	BDNF_IX	27679923	-	0.756	0.961	0.341
chr11	BDNF_IX	27679977	-	0.422	0.814	-0.563
chr11	BDNF_IX	27680000	-	**0.011**	0.247	1.522
chr11	BDNF_IX	27680033	-	0.064	0.497	0.714
chr11	BDNF_IV1	27721663	-	0.620	0.905	0.100
chr11	BDNF_IV1	27721665	-	0.508	0.832	0.133
chr11	BDNF_IV1	27721669	-	0.061	0.504	-0.158
chr11	BDNF_IV1	27721701	-	0.972	0.990	0.009
chr11	BDNF_IV1	27721714	-	0.162	0.584	-0.158
chr11	BDNF_IV1	27721734	-	**0.034**	0.465	-0.264
chr11	BDNF_IV1	27721742	-	0.858	0.985	-0.077
chr11	BDNF_IV1	27721745	-	0.349	0.753	0.215
chr11	BDNF_IV1	27721748	-	0.133	0.623	-0.568
chr11	BDNF_IV1	27721759	-	0.422	0.828	0.344
chr11	BDNF_IV1	27721767	-	0.742	0.966	-0.019
chr11	BDNF_IV1	27721782	-	**0.017**	0.268	0.533
chr11	BDNF_IV1	27721792	-	0.513	0.826	0.220
chr11	BDNF_IV1	27721799	-	0.284	0.666	0.245
chr11	BDNF_IV1	27721802	-	0.676	0.948	0.084
chr11	BDNF_IV1	27721804	-	0.275	0.660	-0.224
chr11	BDNF_IV1	27721817	-	0.435	0.796	0.110
chr11	BDNF_IV1	27721820	-	0.940	1.015	-0.001
chr11	BDNF_IV1	27721834	-	0.696	0.928	-0.187
chr11	BDNF_IV2	27722241	-	0.892	0.984	0.060
chr11	BDNF_IV2	27722243	-	0.755	0.971	0.027
chr11	BDNF_IV2	27722249	-	0.254	0.669	0.250
chr11	BDNF_IV2	27722256	-	0.154	0.595	0.225
chr11	BDNF_IV2	27722265	-	0.691	0.945	0.128
chr11	BDNF_IV2	27722278	-	0.444	0.786	0.111
chr11	BDNF_IV2	27722292	-	0.496	0.837	0.120
chr11	BDNF_IV2	27722296	-	0.091	0.581	0.133
chr11	BDNF_IV2	27722298	-	0.090	0.611	0.110
chr11	BDNF_IV2	27722304	-	0.211	0.632	0.105
chr11	BDNF_IV2	27722334	-	0.692	0.934	0.041
chr11	BDNF_IV2	27722344	-	0.959	0.987	-0.023
chr11	BDNF_IV2	27722346	-	0.157	0.585	0.157
chr11	BDNF_IV2	27722351	-	0.715	0.942	0.013
chr11	BDNF_IV2	27722353	-	0.634	0.912	0.119
chr11	BDNF_IV2	27722371	-	0.947	1.012	0.060

chr11	BDNF_IV2	27722381	-	0.265	0.666	-0.031
chr11	BDNF_IV2	27722389	-	0.615	0.910	0.065
chr11	BDNF_IV2	27722399	-	0.483	0.828	-0.038
chr11	BDNF_IV2	27722410	-	0.577	0.903	0.144
chr11	BDNF_IV2	27722414	-	0.661	0.939	0.088
chr11	BDNF_IV2	27722419	-	0.852	0.990	0.050
chr11	BDNF_IV2	27722427	-	0.875	0.974	0.026
chr11	BDNF_IV3	27723126	-	0.234	0.649	0.133
chr11	BDNF_IV3	27723129	-	0.234	0.665	0.137
chr11	BDNF_IV3	27723130	-	0.174	0.569	1.103
chr11	BDNF_IV3	27723138	-	0.174	0.587	0.147
chr11	BDNF_IV3	27723144	-	0.958	0.995	0.003
chr11	BDNF_IV3	27723160	-	0.921	1.004	-0.005
chr11	BDNF_IV3	27723162	-	0.792	0.961	0.020
chr11	BDNF_IV3	27723191	-	0.599	0.898	0.028
chr11	BDNF_IV3	27723204	-	0.364	0.756	0.125
chr11	BDNF_IV3	27723215	-	0.303	0.682	0.304
chr11	BDNF_IV3	27723219	-	0.442	0.796	0.365
chr11	BDNF_IV3	27723238	-	0.150	0.625	0.810
chr11	BDNF_IV3	27723246	-	0.134	0.601	0.373
chr11	BDNF_IV3	27723267	-	0.131	0.642	0.598
chr11	BDNF_IV3	27723291	-	0.273	0.669	0.406
chr11	BDNF_IV3	27723328	-	0.254	0.654	0.128
chr11	BDNF_I1	27743474	-	0.790	0.969	0.198
chr11	BDNF_I1	27743477	-	0.871	0.980	0.079
chr11	BDNF_I1	27743489	-	0.814	0.977	-0.084
chr11	BDNF_I1	27743494	-	0.950	1.006	-0.001
chr11	BDNF_I1	27743510	-	0.575	0.913	0.111
chr11	BDNF_I1	27743529	-	0.183	0.581	0.659
chr11	BDNF_I1	27743547	-	0.403	0.821	0.286
chr11	BDNF_I1	27743556	-	1.000	1.000	0.003
chr11	BDNF_I1	27743581	-	0.093	0.560	0.445
chr11	BDNF_I1	27743584	-	0.426	0.794	0.231
chr11	BDNF_I1	27743595	-	0.353	0.748	0.247
chr11	BDNF_I1	27743620	-	0.690	0.955	0.028
chr11	BDNF_I1	27743643	-	0.162	0.566	0.107
chr11	BDNF_I1	27743649	-	0.837	0.983	0.021
chr11	BDNF_I1	27743652	-	0.586	0.904	0.054
chr11	BDNF_I1	27743655	-	0.457	0.796	0.085
chr11	BDNF_I1	27743665	-	0.419	0.837	0.106
chr11	BDNF_I1	27743674	-	0.130	0.671	0.504
chr11	BDNF_I1	27743680	-	0.249	0.672	0.451
chr11	BDNF_I1	27743730	-	0.982	0.991	-0.008
chr11	BDNF_I2	27744280	-	0.768	0.953	0.041
chr11	BDNF_I2	27744287	-	0.423	0.801	0.222
chr11	BDNF_I2	27744291	-	0.224	0.654	-0.259

chr11	BDNF_I2	27744293	-	0.957	1.003	0.003
chr11	BDNF_I2	27744313	-	**0.047**	0.426	-0.482
chr11	BDNF_I2	27744327	-	0.094	0.534	-0.517
chr11	BDNF_I2	27744338	-	**0.040**	0.393	0.224
chr11	BDNF_I2	27744346	-	0.587	0.893	0.141
chr11	BDNF_I2	27744364	-	0.501	0.832	-0.143
chr11	BDNF_I2	27744456	-	**0.000**	**0.008**	-0.348
chr11	BDNF_I2	27744464	-	0.146	0.631	-0.134
chr11	BDNF_I2	27744467	-	**0.003**	0.158	-0.704
chr11	BDNF_I2	27744474	-	0.864	0.982	0.187
chr11	BDNF_I2	27744476	-	**0.003**	0.114	0.544
chr11	BDNF_I2	27744477	-	**0.035**	0.421	0.580
chr11	BDNF_I2	27744491	-	0.151	0.602	-0.184
chr11	BDNF_I2	27744497	-	0.834	0.990	-0.041
chr11	BDNF_I2	27744506	-	**0.016**	0.283	-0.220
chr11	BDNF_I2	27744525	-	0.301	0.691	0.051
chr11	BDNF_I2	27744538	-	0.342	0.754	0.029
chr11	BDNF_I2	27744558	-	0.071	0.514	-0.627
chr11	BDNF_I2	27744564	-	**0.004**	0.116	-1.428

p-values in bold are statistically significant. *FDR correction

Supplementary Table S5. Spearman correlation between methylation of *BDNF* amplicons and *BDNF* gene expression

Amplicon	*BDNF* gene expression ($2^{\wedge\text{-ddCt}}$)
BDNF_IX	ρ=0.011; p=0.921
BDNF_IV1	ρ=-0.119; p=0.268
BDNF_IV2	ρ=-0.149; p=0.173
BDNF_IV3	ρ=0.029; p=0.793
BDNF_I1	ρ=-0.072; p=0.516
BDNF_I2	ρ=-0.020; p=0.862

Relative *BDNF* gene expression was calculated as $2^{\wedge\text{-ddCt}}$. BDNF - Brain-Derived Neurotrophic Factor

Supplementary Table S6. BDNF plasma concentration (ng/ml) in participants with MCI and dementia

MCI	Dementia
0.115	0.090
0.092	0.098
0.111	0.110
0.138	0.117
0.133	0.113
0.134	0.110
0.138	0.093
0.083	0.123
0.105	0.130
0.083	0.121
0.105	0.140
0.127	0.091

0.130	0.160
0.137	0.093
0.095	0.108
0.108	0.115
0.122	3.199
0.128	0.282
0.113	0.163
0.104	0.126
0.119	0.092
0.117	0.183
0.132	0.110
0.129	0.116
0.085	0.123
0.135	0.199
0.121	0.205
0.138	0.191
0.124	0.250
0.092	0.324
0.126	0.297
0.164	0.289
0.092	0.215
0.217	0.201
0.127	0.279
0.198	0.335
0.185	0.283
0.206	0.303
0.127	0.246
0.140	0.287
0.145	0.554
0.115	0.402
0.136	0.277
0.150	0.377
0.196	0.287
0.153	0.330
0.209	0.312
0.250	0.231
0.200	0.705
0.222	0.281
0.202	0.315
0.190	0.498
0.215	1.411
0.370	1.048
0.227	0.593
0.309	0.552
0.263	1.230
0.276	5.980
0.202	0.450
0.199	0.459

0.210	0.966
0.231	0.586
0.200	0.505
0.321	0.438
0.211	0.534
0.201	0.880
0.238	0.483
0.345	0.065
0.212	0.117
0.216	0.063
0.215	0.063
0.220	0.069
0.234	1.656
0.265	0.140
0.259	0.128
0.257	0.145
0.222	0.079
0.301	0.078
0.271	0.170
0.318	0.214
0.437	0.094
0.451	0.118
0.404	0.123
0.820	0.075
0.480	0.128
0.331	0.147
0.182	0.129
0.321	0.190
0.456	0.118
0.463	0.126
0.319	0.117
0.278	0.083
0.229	0.141
0.186	0.130
0.246	0.164
0.488	0.166
0.374	0.140
0.253	0.076
0.314	0.077
0.221	0.106
0.453	0.090
0.571	0.075
0.353	0.095
0.285	0.099
0.612	0.131
0.537	0.110
0.526	0.187
1.106	0.157

1.234	0.128
1.048	0.096
1.328	0.126
1.355	0.130
1.247	0.137
1.168	0.088
2.291	0.103
1.078	0.140
0.910	0.143
1.111	0.167
0.293	0.102
1.286	0.112
1.522	0.088
0.124	0.086
1.078	0.112
0.943	0.146
0.448	0.168
1.134	0.091
1.291	0.092
1.353	0.186
1.150	0.111
1.014	0.114
0.523	0.123
1.349	0.102
1.120	0.195
1.221	0.090
1.015	0.224
0.775	0.166
0.866	0.106
0.733	0.174
1.296	0.194
0.433	0.118
1.522	0.266
4.128	
0.473	
1.685	
0.810	
0.446	
0.475	
0.487	
0.606	
0.469	
0.447	
0.693	
0.479	
0.489	
0.475	
0.696	

0.448
0.433
0.669
0.497
0.703
0.708
0.524
1.110
0.762
1.625
0.911
0.530
0.475
0.081
0.080
0.082
0.077
0.066
0.108
0.081
0.167
0.066
0.412
0.063
0.089
0.135
0.063
0.067
0.088
0.120
0.096
0.126
0.164
0.091
0.117
0.088
0.125
0.063
0.615
0.119
0.155
0.145
0.160
0.129
0.118
0.138
0.440
0.156

0.742	
0.109	
0.141	
0.188	
0.138	
0.179	
0.190	
0.175	
0.190	
0.102	
0.244	
0.186	
0.195	
0.108	
0.094	
0.095	
0.107	
0.136	
0.146	
0.187	
0.191	
0.078	
0.078	
0.132	
0.139	
0.090	
0.084	
0.075	
0.113	
0.211 (0.127-0.477) *	0.140 (0.110-0.280) *

*Data represent median (25th - 75th percentile) with outliers excluded. Plasma BDNF concentrations were quantified using commercially available sandwich enzyme-linked immunosorbent assay (ELISA) kit (ELK5404). BDNF - Brain-Derived Neurotrophic Factor, MCI – Mild Cognitive Impairment
